# Real-World Comparison of FFR and QFR: New Perspectives on the Functional Assessment of Coronary Stenoses

**DOI:** 10.3390/jcm14175946

**Published:** 2025-08-22

**Authors:** Róbert Gál, Bettina Csanádi, Tamás Ferenci, Noémi Bora, Zsolt Piróth

**Affiliations:** 1 Gottsegen National Cardiovascular Center, 1096 Budapest, Hungary; robert.gal2@gokvi.hu (R.G.); bettina.csanadi@gokvi.hu (B.C.); noemi.bora@gokvi.hu (N.B.); 2 Károly Rácz Doctoral School of Clinical Medicine, Semmelweis University, 1085 Budapest, Hungary; 3 Physiological Controls Group John von Neumann Faculty of Informatics, Obuda University, 1034 Budapest, Hungary; ferenci.tamas@nik.uni-obuda.hu; 4 Department of Statistics Corvinus, University of Budapest, 1093 Budapest, Hungary

**Keywords:** FFR, QFR, bias, learning curve

## Abstract

**Background/Objectives:** The diagnostic value of Quantitative Flow Ratio (QFR) with respect to Fractional Flow Reserve (FFR) in real-world settings is not well described, and neither are the factors influencing the bias of QFR versus FFR well understood. The learning curve associated with QFR calculation has not been thoroughly investigated. Hence, we sought to evaluate the association between the QFR and FFR, to investigate the influence of clinical parameters on both values and their difference, and to analyze the learning curve associated with QFR measurement in a real-world setting. **Methods**: All patients who underwent FFR and QFR measurements in 2023 at our tertiary-care center were included. The bias was characterized using a Bland–Altman plot and multivariable regression was used to uncover its potential predictors. **Results**: QFR calculation was successful in 73% of 595 patients with 778 vessels with FFR measurement results. Median bias of QFR was 0.011, but in 7% of the cases, the difference between the two exceeded 0.10. A good correlation was found between the two indices. Receiver operating characteristic curve analysis showed that the area under the curve of QFR for predicting FFR ≤ 0.80 was 0.912. FFR and QFR values were lower in the left anterior descending artery; acute coronary syndrome indication was associated with higher QFR values. Right coronary artery localization was associated with a greater bias of QFR, whereas female gender and aortic stenosis were associated with a lower bias of QFR. Both measurement time and bias decreased in a non-linear fashion with increasing experience. **Conclusions**: Clinical and angiographic factors affect the bias of QFR versus FFR. QFR has a short learning curve with growing experience leading to shorter measurement time and less bias.

## 1. Introduction

Coronary angiography remains the gold standard for diagnosing coronary artery disease (CAD), nevertheless its inherent limitations pose considerable challenges. To address these limitations, coronary physiological measurements such as fractional flow reserve (FFR) have been applied. Multiple large-scale clinical trials [1,2] have demonstrated the superiority of FFR-guided revascularization over angiography-based strategies; however, the invasive nature of FFR—requiring the induction of hyperemia and the application of a pressure-wire—along with associated costs, have contributed to its limited adoption [3,4].

Quantitative flow ratio (QFR), an emerging coronary physiological index, offers a non-invasive alternative to FFR by leveraging computational fluid dynamics (CFD) to assess the functional significance of stenoses [5]. QFR analysis requires at least two angiographic projections of the target vessel, with a minimum angular difference of 25°, minimal overlap, and adequate contrast filling of the vessel. Similar to FFR, QFR values range from 0 to 1, with a clinical cutoff of 0.80. Its advantages include a less invasive approach, and potentially lower costs compared to FFR. Theoretically, QFR can be calculated offline, any time after the angiogram is completed, or in real time, during the invasive procedure, allowing decisions regarding revascularization to be made based on QFR. For the latter to be realistic, the calculation should not take more than a few minutes, and the length of the procedure should be comparable to that of the FFR measurement. However, the accuracy of QFR is highly dependent on angiographic image quality and, probably, operator expertise as well [6]. Studies such as FAVOR Pilot, FAVOR II: China, and FAVOR II: Europe-Japan [7,8,9] have confirmed a strong correlation and agreement between QFR and FFR, supporting its diagnostic performance. Additionally, the multicenter, randomized FAVOR III: China trial [10] demonstrated that QFR-guided revascularization yielded superior clinical outcomes compared to angiography-guided strategies, reinforcing its clinical applicability. As a result, the 2024 European Society of Cardiology (ESC) guidelines [11] recommend the use of QFR alongside FFR for evaluating coronary stenoses in chronic coronary syndromes. However, despite promising results in angiography-based comparisons, the FAVOR III: Europe study [12] concluded that QFR had inferior diagnostic value compared to FFR-guided evaluation.

Despite these advances, the agreement between QFR and FFR outside of research settings remains unclear. Additionally, factors influencing QFR value and its deviation from FFR have yet to be fully elucidated. Notably, the learning curve, in terms of accuracy and length of QFR calculation has not been thoroughly investigated.

Therefore, the aim of the present study is to evaluate the association between QFR and FFR, to investigate the influence of clinical parameters on both values and their difference, and to analyze the learning curve associated with QFR measurement in a real-world setting.

## 2. Materials and Methods

### 2.1. Patients

All patients who underwent clinically indicated FFR assessment of at least one coronary artery at the Gottsegen National Cardiovascular Center between 1 January 2023 and 31 December 2023, followed by a successful offline QFR calculation were retrospectively enrolled in our study. No restrictions were applied concerning the indication for coronary angiography. In cases of acute coronary syndrome (ACS), measurements were conducted in the non-culprit vessels. All patients gave written informed consent before the procedure. The study was conducted in accordance with the Declaration of Helsinki.

### 2.2. FFR Measurement

Invasive coronary angiography was carried out according to current best practices. FFR measurements were performed using commercially available pressure wires (St. Jude Medical, now Abbott, St. Paul, MN, USA). Hyperemia was induced using intracoronary boluses of adenosine—200 μg for the left coronary artery and 100 μg for the right, or by intravenous adenosine infusion at a standard dose of 140 μg/kg/min. After the procedure, all patients received medical therapy in accordance with current clinical guidelines.

### 2.3. QFR Calculation

The measurements were conducted by an examiner (RG) with no prior experience in QFR calculation or coronary angiography, utilizing the Medis Suite 4.0.62.4 software (Medis Medical Imaging Systems BV, Leiden, The Netherlands). The examiner was blinded to the FFR values. QFR analysis was performed without adherence to a standardized angiographic acquisition protocol; in fact, routine coronary angiograms were utilized. Two end-diastolic angiographic frames, acquired from projections at least 25 degrees apart, were used to construct a 3D model of the coronary artery, ensuring minimal vessel foreshortening and overlap. The path line for QFR calculation started at the vessel ostium and ended at the location of the pressure wire sensor. Measurement time was recorded for each calculation, defined as the interval from the completion of loading the coronary angiographic images to the appearance of the QFR value on the screen.

### 2.4. Statistical Analysis

Continuous variables are presented as median (lower quartile—upper quartile), categorical variables are presented as count (proportion).

The agreement between QFR and FFR was visualized using Bland–Altman plot and characterized by limits of agreement.

To characterize the learning curve, we assessed changes in accuracy (defined as the absolute difference between FFR and QFR values) and measurement time using multivariable regression to control for other factors that might have changed over time. Regressions employed a robust covariance matrix estimation to account for clustering of measurements coming from the same patient.

Calculations were carried out under R statistical program package version 4.5.0.

## 3. Results

From 1 January 2023 to 31 December 2023, 595 patients with 778 vessels were included. QFR analysis was attempted for all cases, with successful calculations in 73% of cases (568 vessels in 435 patients).

**Figure 1:**
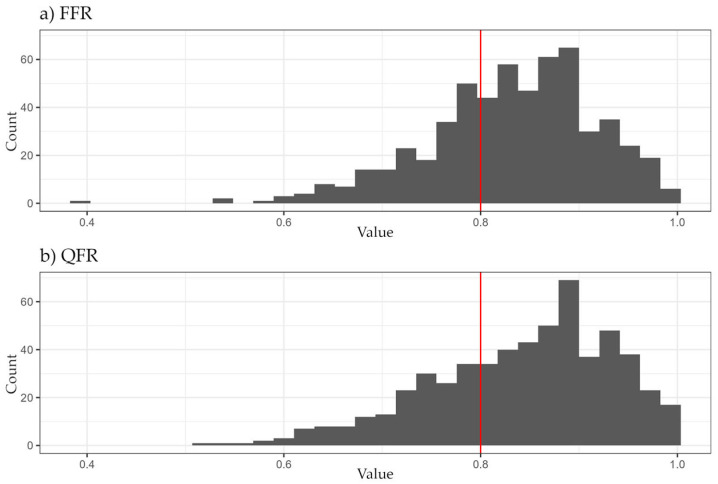
Distribution of (**a**) FFR and (**b**) QFR values. FFR: Fractional Flow Reserve; QFR: Quantitative Flow Ratio.

Baseline clinical and vessel characteristics are listed in Table 1. Median FFR and QFR values, absolute differences, and measurement times are listed in Table 2. The distribution of FFR and QFR values can be seen in Figure 1.

### 3.1. Causes of QFR Calculation Failure

The most frequent cause of failure of QFR calculation was the absence of two angiographic projections with a minimum angular separation of 25° (90 cases; 43%). Excessive vessel overlap was observed in 66 cases (31%), while insufficient contrast-filling prevented QFR assessment in 34 cases (16%). Additionally, inadequate image quality precluded calculation in 20 cases (10%).

### 3.2. Correlation and Agreement

**Figure 2:**
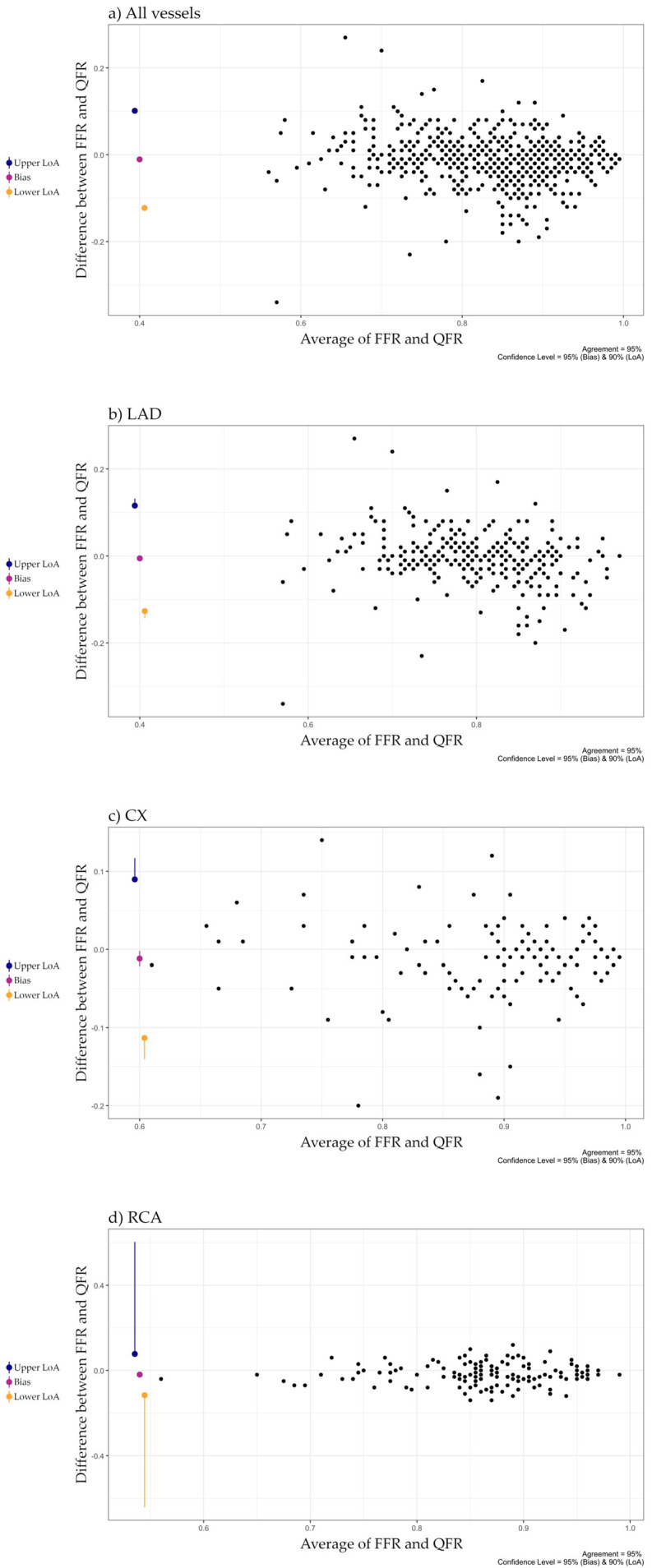
Agreement between FFR and QFR in (**a**) all vessels, (**b**) LAD, (**c**) Cx, (**d**) RCA LAD: Left Anterior Descending Artery; Cx: Left Circumflex Artery; RCA: Right Coronary Artery.

**Figure 3:**
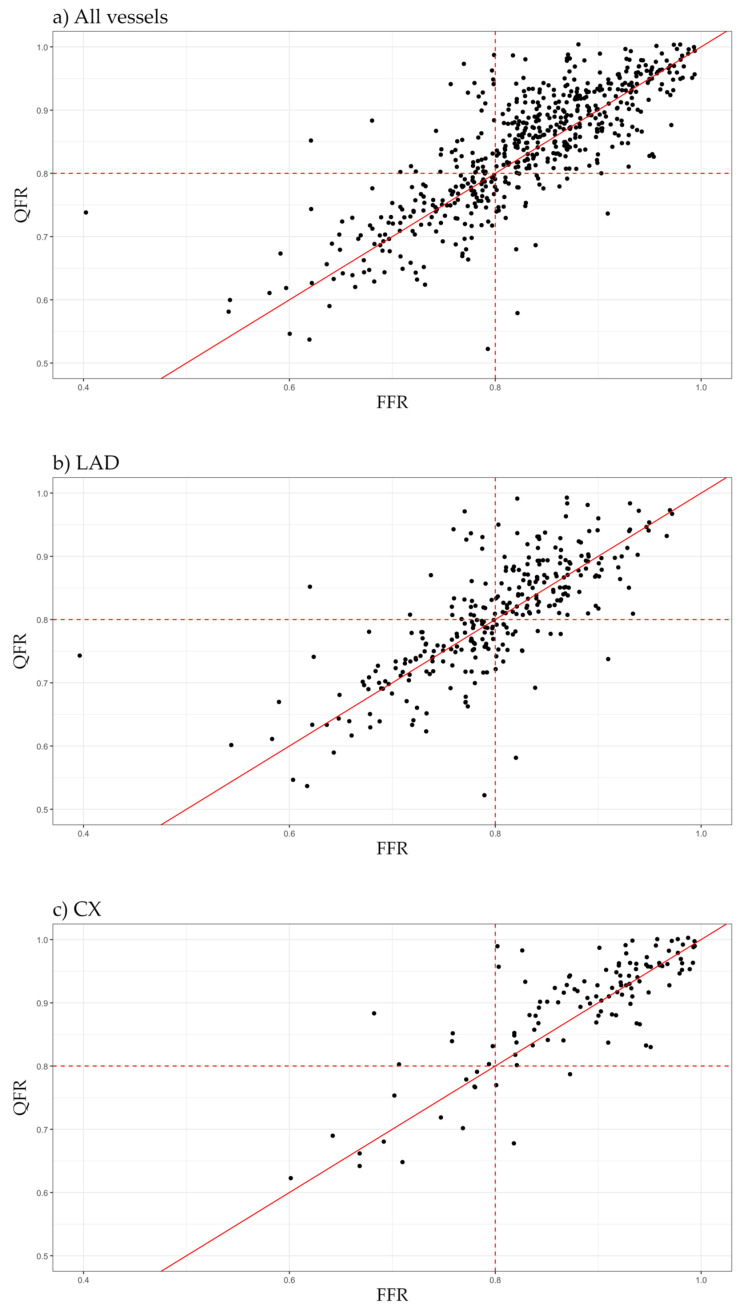

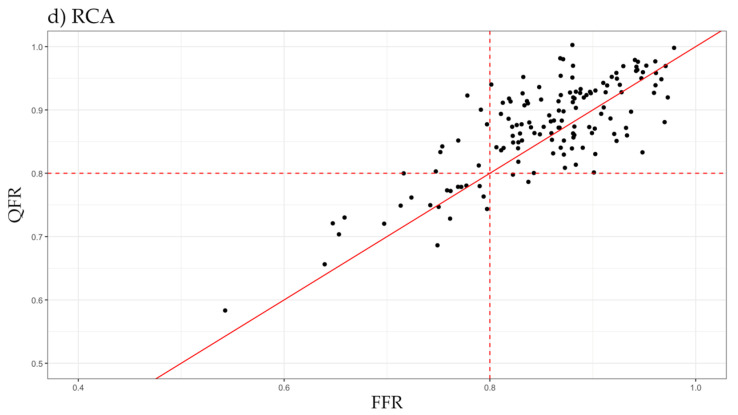
Correlation between FFR and QFR in (**a**) all vessels, (**b**) LAD, (**c**) Cx, (**d**) RCA.

A good agreement was observed between FFR and QFR with a bias of 0.011. The limits of agreement were −0.123 and 0.101. Per-vessel analysis showed best agreement in the left anterior descending artery (LAD, bias: 0.006; limits of agreement: −0.127, 0.116), worst in the right coronary artery (RCA, bias: −0.019; limits of agreement: −0.116, 0.078). For the left circumflex artery (Cx) mean difference was −0.012, with limits of agreement of −0.113 and 0.090. The absolute difference between the values exceeded 0.05 in 144 cases (25%), and 0.10 in 37 cases (7%). Good correlation was found between the two indices (r = 0.802). The correlation coefficient was highest in LAD (0.775), lowest in RCA (0.675) and 0.765 in the Cx. The Bland–Altman plots and scatterplots are shown in Figure 2 and Figure 3.

### 3.3. Diagnostic Performance of QFR

The confusion matrix comparing FFR and QFR with a cut-off of 0.8 can be seen in Table 3.

**Figure 4:**
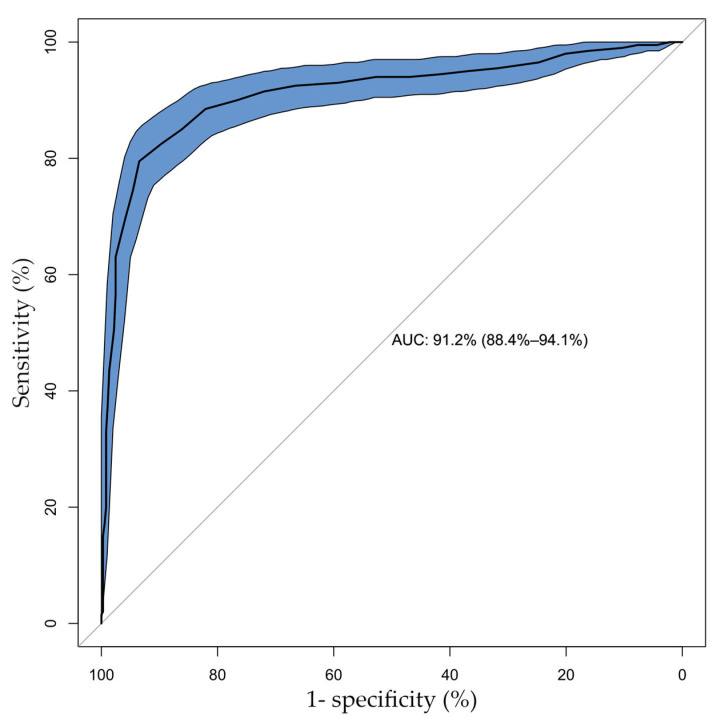
Diagnostic performance of QFR with respect to FFR.

Using FFR ≤ 0.80 as reference, the sensitivity, specificity, positive and negative predictive values of QFR were 80% (95% CI, 0.73–0.85), 93% (95% CI, 0.90–0.96), 87% (95% CI, 0.81–0.91), and 89% (95% CI, 0.86–0.92), respectively. Furthermore, receiver operating characteristic curve analysis showed that the area under the curve (AUC) of QFR for predicting FFR ≤ 0.80 was 0.912 (95% CI, 0.884–0.941), shown in Figure 4.

### 3.4. Predictors of FFR and QFR

**Figure 5:**
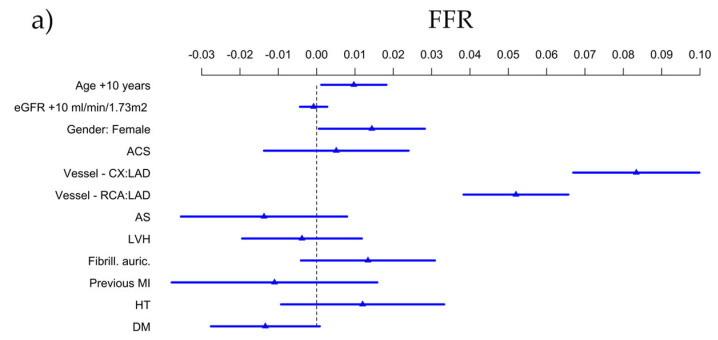

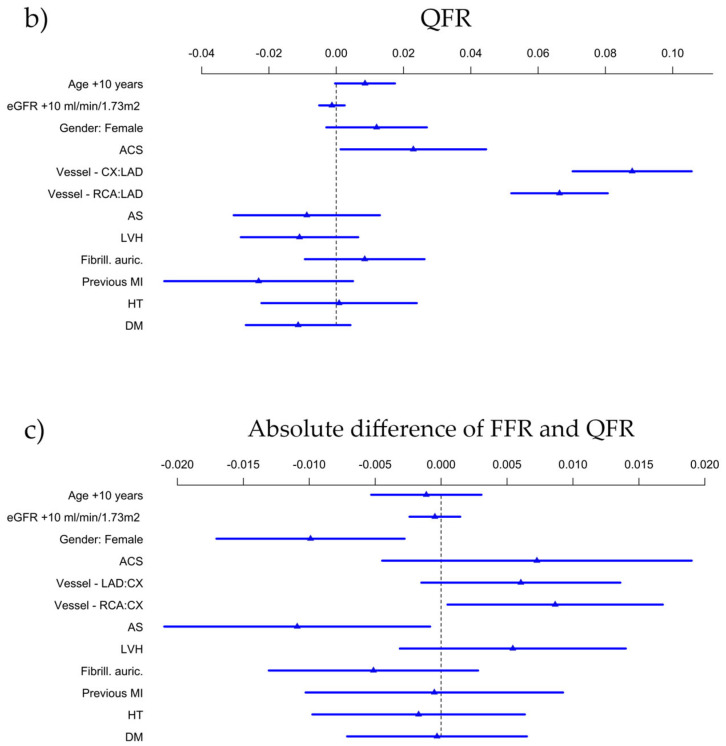
Predictors of (**a**) FFR, (**b**) QFR, (**c**) absolute difference of FFR and QFR.

The effect of clinical parameters on the indices is shown in Figure 5.

For FFR, LAD was found to be a significant negative predictor (*p* < 0.001). Moreover, advanced age (*p* = 0.025) and female sex (*p* = 0.041) resulted in significantly higher values (shown in Figure 5a).

LAD localization was also associated with significantly lower QFR values (*p* < 0.001), while acute coronary syndrome indication was a predictor of higher QFR values (*p* = 0.038), as illustrated in Figure 5b.

**Figure 6:**
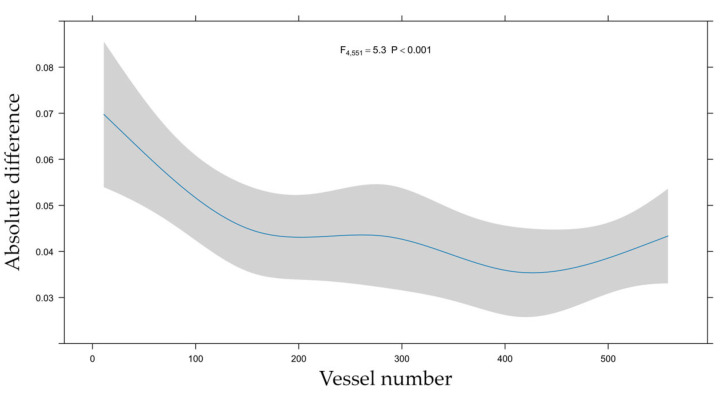
Learning curve of QFR calculations—accuracy. This graph shows how the absolute difference between FFR and the calculated QFR (Y-axis) changed with increasing experience, i.e., growing number of calculations performed (X-axis).

The absolute difference between QFR and FFR was greater in the RCA than in the Cx (*p* = 0.038) and tended to be greater in the Cx than in the LAD, but this did not reach statistical significance, whereas it was smaller in female patients (*p* = 0.006) and in patients with aortic stenosis (*p* = 0.033), as shown in Figure 5c. The effect of the observer experience on the absolute bias of QFR with respect to FFR was found to be non-linear and is illustrated in Figure 6.

### 3.5. Learning Curve

**Figure 7:**
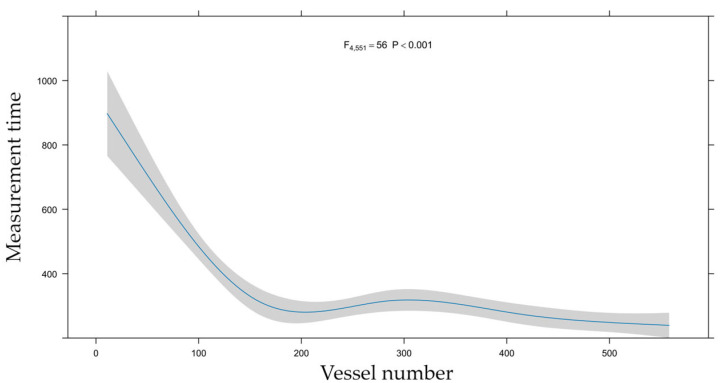
Learning curve of QFR calculations—measurement time. This graph shows how the measurement time of QFR (Y-axis) changed with increasing experience, i.e., growing number of calculations performed (X-axis).

Measurement time decreased drastically with increasing experience. The non-linear relationship can be seen in Figure 7.

Median measurement time for QFR calculation was 271 s (IQR: 206, 374), shortest in RCA, 236 s (IQR: 191, 329), and longest in LAD, 302 s (IQR: 222, 437), while 257 s (IQR: 211, 333) in Cx (Table 2). The median measurement time in the first 100 calculations was 560 s (IQR: 418, 719) and 226 s (IQR: 168, 251) in the last 100. The effect of growing experience could also be tracked in measurement accuracy: in the first 100 measurements, the median absolute bias of QFR with respect to FFR was 0.05, while only 0.03 in the last 100 (Figure 6).

## 4. Discussion

In this study, we evaluated the diagnostic performance of offline QFR calculation compared to the gold-standard pressure wire-derived FFR in an all-comers setting and assessed clinical predictors influencing these indices and their discrepancies. Additionally, we analyzed the learning curve associated with QFR calculations. Our salient findings are as follows.

Offline QFR calculation was unsuccessful in 27% of cases, primarily due to the lack of projections with 25° angular difference, vessel overlap, inadequate contrast enhancement, or suboptimal image quality.

QFR had a good agreement with FFR, although individual deviations resulted in significant differences in clinical decision making.

FFR and QFR values were lower in the left anterior descending artery, while female sex and advanced age were associated with higher FFR values. Acute coronary syndrome indication was linked to higher QFR values. In female patients and those with aortic stenosis, a lower bias of QFR was found, whereas the bias of QFR was greater in the RCA than in the Cx.

Both measurement time and bias decreased in a non-linear fashion with increasing experience and seemed to plateau after about 200 measurements.

The observed 27% failure rate is consistent with prior studies employing retrospective QFR calculations. To improve the success rate, adherence to minimal acquisition standards during coronary angiography is recommended. Most importantly, ensuring two projections with an angular difference of at least 25° between the two views is essential for reliable 3D reconstruction. In addition, vessel-specific angulations should be selected to minimize foreshortening and overlap. Adequate contrast filling, minimal table or patient movement, and avoidance of vessel overlap with catheters or side branches are also key factors. These acquisition principles are in line with existing best practices in diagnostic coronary angiography. Implementing standardized acquisition guidelines may substantially reduce the failure rate and enhance the feasibility and reproducibility of QFR in everyday clinical practice.

Beyond the learning curve analysis, our study adds novel insights by evaluating the predictors of the QFR-FFR bias—a topic that is underexplored in the current literature. We specifically investigated how clinical and anatomical variables, such as vessel type, sex, and aortic stenosis influenced the agreement between QFR and FFR. Additionally, by conducting all measurements in a high-volume center with a single, untrained operator, our study offers real-world data on the practical feasibility of QFR implementation in routine workflows, even without formalized training structures. These elements aim to complement existing validation studies and clinical trials by addressing specific gaps related to usability, limitations, and performance of QFR in various clinical settings.

### 4.1. Comparison of QFR and FFR

Despite a strong correlation, the observed discrepancy between QFR and FFR was statistically and clinically significant. The median difference between QFR and FFR was 0.01, indicating a consistent underestimation of stenosis severity by QFR. This pattern was evident across all three coronary arteries but was most pronounced in the RCA, where QFR values exceeded FFR by 0.02.

Notably, in some cases, QFR exhibited considerable deviation from FFR. In our cohort, the absolute difference between QFR and FFR exceeded 0.05 in 144 cases (25.4%) and 0.10 in 37 cases (6.5%), findings comparable to the FAVOR validation studies [7,8,9,10].

The clinical significance of these discrepancies is highlighted by the fact that in 11% of cases, binary QFR stenosis classification (significant/non-significant) was different from FFR classification, leading to discordant clinical decisions. According to our data, in 63% of discordant cases, QFR underestimated stenosis severity. Since clinical decisions in our study were based on FFR, such lesions would have been missed by QFR, potentially leaving functionally significant stenoses untreated. Conversely, in 37% of discordant cases, QFR overestimated lesion severity, exposing patients to the risks of dual antiplatelet therapy, procedural, and stent-related complications such as in-stent restenosis or stent thrombosis—without clear objective evidence of myocardial ischemia. Our findings align with large-scale studies, reporting a diagnostic accuracy of 86–93% in the FAVOR trials [7,8,9,10].

Despite these differences, QFR-guided interventions have demonstrated superior clinical outcomes compared to angiography alone [10]. Since in the FAVOR III: Europe study, QFR could not meet non-inferiority compared to FFR, it can be positioned between angiography and FFR in terms of clinical decision making [12]. In settings where FFR is not available, QFR represents a viable alternative.

In the QFR range of 0.75–0.85, only 78% of binary clinical decisions were similar based on FFR and QFR. Westra et al. proposed a hybrid decision making algorithm, integrating both QFR and FFR. In this approach, an initial QFR assessment is performed, if the result falls outside a predefined “gray zone,” treatment decisions are based solely on QFR, while values in the gray zone necessitate confirmatory FFR measurements [9]. This strategy reduces costs, mitigates risks associated with invasive FFR assessment, and minimizes decision making errors arising from discrepancies of FFR and QFR. Using a cutoff range of 0.75–0.85, in our study, 66% of FFR measurements could have been avoided, whilst improving diagnostic accuracy to 94% in cases of QFR values outside of this range. However, there is currently no clinical evidence supporting the clinical application of this hybrid algorithm, and its proposed lower and upper cutoff values.

### 4.2. Predictors of FFR, QFR and Their Difference

Our analysis identified LAD localization as a significant negative predictor of FFR, consistent with prior research. Several factors may contribute to this observation. One straightforward explanation is the effect of hydrostatic pressure [13]: in the supine position, the distal LAD is positioned higher relative to the ostium of the left main coronary artery, altering the Pd/Pa ratio. Additionally, LAD-specific characteristics, such as greater prevalence of atherosclerosis and elevated diastolic flow velocities, contribute to lower FFR values [14,15]. Notably, the ratio of intravascular volume to myocardial mass is lowest in the LAD, further influencing flow dynamics [16]. Among clinical parameters, age demonstrated a significant correlation with FFR, in agreement with previous studies [17]. This relationship may be attributed to the progressive development of macrovascular and microvascular dysfunction with aging, leading to impaired response to adenosine. Male sex was identified as a significant predictor of lower FFR values. This observation aligns with the well-documented higher prevalence of atherosclerotic cardiovascular disease among men compared to women, particularly in middle-aged populations—a disparity attributed to sex-related differences in vascular biology, endothelial function, exposure to cardiovascular risk factors; and larger subtended myocardial mass [18,19]. Although diabetes mellitus was not found to be significantly associated with lower FFR, a clear trend was observed, likely reflecting the diffuse, severe atherosclerotic burden characteristic of diabetic patients.

In the QFR analysis, in addition to LAD localization, measurements performed in the setting of acute coronary syndrome emerged as a significant predictor of QFR values. Of note, in ACS, FFR and QFR measurements were performed in the non-culprit vessels. The influence of ACS indication warrants particular attention. Approximately half of the patients presenting with ST-elevation myocardial infarction have angiographically multivessel disease, and FFR-guided complete revascularization was found to be beneficial compared to medical therapy alone of the culprit lesions in two randomized clinical studies [20,21]. Moreover, in a post hoc analysis of the COMPARE-ACUTE trial, FFR measured in the non-culprit vessels treated medically (without revascularization) immediately after successful primary PCI was found to have significant inverse relationship with vessel-related major adverse events [22], proving its prognostic value. However, FFR is infrequently measured in the non-culprit arteries in the acute setting, and angiography is known to overestimate the significance of bystander disease when compared to repeat angiography later on [23]. Thus, there is an unmet need of the practicing clinician for a reliable, readily available tool for decision making in terms of non-culprit revascularization in acute coronary syndrome. Such a tool could be an angiography-based physiology index with the potential of optimizing workflow in the catheterization laboratory, provided its accuracy is unaffected by acute coronary syndrome.

In the present study, ACS was associated not only with significantly higher QFR values but also with a greater discrepancy between QFR and the corresponding FFR values. While the median bias of QFR vs. FFR in chronic coronary syndrome cases was 0.01, this bias was found to be 0.03 in ACS measurements, suggesting that QFR may substantially underestimate the functional severity of coronary stenoses of non-culprit lesions in the acute setting. This increased inaccuracy may be related to changes in microcirculation during acute coronary syndrome. On the other hand, it is important to emphasize that in multivariable analysis, ACS was not found to be an independent predictor of greater inaccuracy of QFR versus FFR in our study (Figure 5c). Given the limited number of measurements presented herein, further studies are required to investigate these observations in greater detail. Of note, a post hoc analysis of the Intravascular Ultrasound Guided PCI in STEMI (iSTEMI) trial concluded that QFR calculated from the acute-phase angiogram had a very good diagnostic performance with staged FFR as reference [24]. In another retrospective, multicenter, observational study, QFR was found to have excellent diagnostic accuracy in the non-culprit vessels during primary PCI [25].

QFR demonstrated significantly greater accuracy in female patients and in those with concomitant aortic stenosis. In severe aortic stenosis, the altered aortic flow pattern can lower the coronary translesional pressure gradient, and contrast flow in the coronary arteries. Since QFR relies heavily on contrast flow, these hemodynamic changes may impact the validity of QFR computation. Interestingly, in our dataset, aortic stenosis was associated with a smaller bias between FFR and QFR. One possible explanation is that the typically slower and potentially more stable coronary flow in aortic stenosis allows for more accurate frame-based velocity estimation, as QFR calculations are derived from contrast propagation at a fixed frame rate. In any case, it is reassuring that the accuracy of QFR is at least as good in aortic stenosis as in other clinical scenarios. This is of particular interest since the prevalence of significant coronary artery disease in patients undergoing surgical aortic valve replacement is around 40%, whereas in cohorts undergoing transcatheter aortic valve implant, it is estimated around 60% without firm evidence that concomitant revascularization is beneficial [26]. The QFR Guided Revascularization Strategy for Patients Undergoing Primary Valve Surgery With Comorbid Coronary Artery Disease (FAVOR4-QVAS) trial (ClinicalTrials.gov ID NCT03977129) is currently comparing the effectiveness of a QFR-based revascularization decision in patients with comorbid coronary artery lesions defined as diameter stenosis of ≥50% (by visual estimation) undergoing surgical aortic valve replacement with a coronary angiography-based approach in preventing the 30-day post-surgical occurrence of a composite of all-cause death, myocardial infarction, stroke, unplanned coronary revascularization, and new renal failure requiring dialysis.

In our dataset, QFR assessments in the right coronary artery exhibited larger absolute deviations from FFR compared to other coronary territories. The poor correlation and agreement between RCA lesions may be attributable to the limited availability of optimal angiographic projections. At our center, for RCA angiography, typically a lateral and a left cranial oblique projection are used. In most cases, the lateral projection could be used for RCA QFR calculation which is non-ideal due to significant foreshortening of the proximal artery segment. This observation underscores the importance of optimal projections for the calculation of QFR. It remains to be seen if calculation algorithms based on a single, good-quality projection (µQFR) share this observation, so far, the published experience with µQFR is limited [27].

Given that QFR is highly dependent on flow dynamics, atrial fibrillation was also considered as a potential predictor of the absolute bias, as it causes irregular cardiac cycle length and variable diastolic duration, which may lead to fluctuations in coronary flow. This variability could theoretically compromise both the feasibility and accuracy of QFR by introducing inconsistency in flow assessment and complicating the selection of end-diastolic frames. However, in our analysis, atrial fibrillation had no measurable effect on the agreement between FFR and QFR or on the measurement time. While our study was not specifically powered for subgroup analysis, these findings suggest that QFR may still be feasible in the presence of atrial fibrillation—though further studies are needed to confirm this under varying clinical conditions.

QFR calculation times were significantly longer in LAD lesions, likely due to vessel length and, potentially, the greater prevalence of diffuse atherosclerosis.

### 4.3. Learning Curve Analysis

As depicted in Figure 7, QFR measurement time decreased in two distinct phases: an initial steep reduction, followed by a mild decline. This non-linear relationship can be approximated by a median reduction to one-third of the original measurement time, with most calculations requiring less than four minutes. These times are consistent with those reported in experienced centers [9].

Early reductions in measurement time likely stem from improved proficiency in coronary angiography interpretation. Calculations involve several interactions with the images, including selecting optimal projections, performing offset corrections, identifying vessel course, and delineating contours. As these skills improve, measurement times decrease. Since image interpretation can be refined independently of QFR software use, this initial phase may be influenced by the examiner’s diligence. Later reductions in time likely reflect enhanced familiarity with software functionality, particularly in reference selection and contour refinement.

There is an inherent lower limit to QFR measurement time, dictated by image loading and software processing. The fastest measurement in our study, requiring minimal manual intervention, was completed in 66 s, this can allow real-time decision making.

Accuracy analysis revealed that QFR-FFR discrepancies decreased with increasing experience, primarily due to improved utilization of the software’s subjective functions. The most significant and learnable factor in reducing bias was appropriate reference selection, particularly in cases of diffuse lesions.

Few studies have investigated the learning curve for QFR calculation. A 2022 study assessed interobserver variability among five examiners with different experience levels and intraobserver variability across repeated measurements of 50 coronary arteries [6]. Their findings demonstrated superior QFR-FFR correlation in experienced examiners; these correlation coefficients are comparable to our results. However, differences in methodology, including the use of QFR 2.0 software, limit direct comparison. The study concluded that increased examiner experience significantly improved QFR-FFR agreement, while intraobserver variability analysis demonstrated that learning effects were evident even after a relatively small number of measurements. In our analysis, both accuracy and measurement time improved until approximately 200 measurements.

A 2025 study further examined the learning curve of QFR in a smaller cohort [28]. While analyzing only 54 vessels per examiner, their findings corroborate the trend of decreasing measurement time; however, their use of structured training in both the interpretation of coronary angiography and QFR software prior to the study precludes extrapolation to untrained observers. Additionally, they reported no effect of experience on the accuracy of QFR.

The learning curve observed in our study provides practical insights into the clinical implementation of QFR. Our findings suggest that institutions may be able to introduce QFR analysis even in the absence of formal training programs, as acceptable performance was achievable by an untrained operator relatively early. However, we acknowledge that structured training would likely accelerate proficiency, reduce user-dependent variability, and improve overall reproducibility.

In summary, previous studies have shown that greater observer experience can lead to quicker, more accurate measurements, and the effect of increasing experience can be seen in intra-observer comparisons. Our study proves that accurate QFR calculations can be achieved in a short time frame, even by an untrained examiner and our analysis assessed these changes quantitatively both in terms of accuracy with respect to FFR and time of calculations. It can be speculated that specific training can accelerate the learning process.

### 4.4. Clinical Implications

Our findings highlight important limitations and contextual considerations regarding the clinical applicability of QFR. While QFR shows reasonable agreement with invasive FFR, its accuracy is notably reduced in certain clinical and anatomical scenarios. Specifically, in patients with acute coronary syndrome, QFR systematically overestimated FFR values in non-culprit vessels. Reliance on QFR in this context may lead to the underdiagnosis of physiologically significant lesions. Furthermore, we observed that QFR accuracy was significantly lower in the right coronary artery, due to suboptimal angiographic angles and foreshortening, that may interfere with 3D reconstruction.

Importantly, despite these limitations, QFR still offers clear advantages over visual angiographic assessment alone: as such, QFR may serve as a reasonable alternative when FFR is not available, particularly in patients with chronic coronary syndrome. In higher risk settings—like ACS—or in the absence of adequate images—in our case, in RCA lesions—over-reliance on QFR may increase the chance of inappropriate clinical decision making [29].

An effective and pragmatic approach may be the adoption of a hybrid physiological assessment model, as mentioned before. This strategy has the potential to significantly reduce the number of invasive wire-based assessments, while maintaining high diagnostic agreement and ensuring accurate treatment decisions.

### 4.5. Further Aspects of QFR Analysis

The calculation of QFR can be performed not only before but also after PCI. In the HAWKEYE study, post-PCI QFR was found to have prognostic value with a three-fold higher vessel-oriented composite endpoint (cardiovascular death, myocardial infarction, ischemia-driven target vessel revascularization) in patients with post-PCI QFR ≤ 0.89 calculated offline [30]. Contrary to this, our experience with post-PCI QFR showed that while post-PCI QFR was associated with outcome, it was not found to be an independent predictor of long-term survival free from target vessel failure [31].

Another potential of angiography-based physiology assessment of coronary circulation is the calculation of microcirculatory function (resistance). An angiography-derived index of microcirculatory resistance (IMR_angio_) was demonstrated to have good correlation with the traditional, invasively measured index of microcirculatory resistance (IMR) in STEMI [32]. A-IMR [33] and angio-IMR [34] were also found to exhibit good correlation with IMR in chronic coronary syndrome.

### 4.6. Limitations

As a retrospective study, image acquisition was not optimized for QFR calculation, that resulted in multiple constraints, including the high exclusion rate of vessels or the frequent use of suboptimal angiographic views and image quality for the analysis. These findings remain consistent with previously published data on offline QFR measurement and allow our conclusions to be generalized in real-world settings.

Our study found the poorest QFR-FFR correlation and agreement in RCA lesions. This may be attributable to the limited availability of optimal angiographic projections. In the majority of cases, the lateral projection was used for RCA QFR calculation which is non-ideal due to significant foreshortening of the proximal artery segment.

Additional limitations of this study include its single-center, single-observer design, which may affect generalizability. Furthermore, all measurements were performed using QFR version 2.1; findings may differ with more recent software iterations, which incorporate updated algorithms and improved computational performance with the potential of reducing software-related bias.

A key limitation of this study is that all QFR measurements were performed by a single operator without formal training and no prior experience in QFR calculations and coronary angiography. While this design reflects a realistic scenario for centers adopting QFR for the first time, it limits the generalizability of our findings, particularly in terms of measurement bias. In real-world clinical settings, QFR measurement is performed by users with varying levels of experience and technical skills which can influence the agreement of the values. Operators with greater expertise in coronary angiography can also be expected to achieve more precise measurements. Of note, we found that both accuracy and measurement time improved until approximately 200 measurements, signifying a short learning curve.

## 5. Conclusions

QFR represents a viable alternative for functional coronary assessment in scenarios where FFR is unavailable in a real-world setting. QFR exhibits a short learning curve, allowing accurate measurements even by examiners with no prior experience of analyzing coronary angiograms. Clinical and angiographic factors affect the bias of QFR relative to FFR.

## Figures and Tables

**Table 1:** Patient and vessel characteristics. IQR: Interquartile Range; LAD: Left Anterior Descending Artery; Cx: Left Circumflex Artery; RCA: Right Coronary Artery; eGFR: Estimated Glomerular Filtration Rate.

Patient Characteristics			Vessel Characteristics		
Number of patients; *n*	435		Number of vessels; *n*	568	
Age; years (IQR)	68.5	(61–75)	Type of vessels		
Female sex; *n* (%)	146	(34%)	LAD; *n* (%)	302	(53%)
Hypertension; *n* (%)	363	(83%)	Cx; *n* (%)	120	(21%)
Diabetes mellitus; *n* (%)	170	(39%)	RCA; *n* (%)	146	(26%)
Acute coronary syndrome; *n* (%)	60	(14%)			
Significant aortic stenosis; *n* (%)	48	(11%)	Prior myocardial infarction in the territory; *n* (%)	51	(9%)
eGFR; ml/min/1.73 m^2^ (IQR)	71	(59–84)			
Atrial fibrillation during exam; *n* (%)	69	(12%)			

**Table 2:** Median values of FFR, QFR, absolute difference in FFR and QFR, and measurement time in different vessels. FFR: Fractional Flow Reserve; QFR: Quantitative Flow Ratio.

	FFR	QFR	Absolute Difference	Measurement Time (s)
All vessels	0.84 (0.78, 0.89)	0.85 (0.78, 0.92)	0.03 (0.01, 0.06)	271 (206, 374)
Type of vessels				
LAD	0.81 (0.76, 0.86)	0.81 (0.75, 0.87)	0.03 (0.01, 0.06)	302 (222, 437)
CX	0.91 (0.83, 0.94)	0.92 (0.85, 0.96)	0.03 (0.01, 0.05)	257 (211, 333)
RCA	0.87 (0.82, 0.90)	0.88 (0.84, 0.93)	0.03 (0.02, 0.06)	236 (191, 329)

**Table 3:** Confusion matrix of FFR and QFR.

FFR	QFR		Total
	≤0.80	>0.80	
≤0.80	159	41	200
>0.80	24	344	368
Total	183	385	568

## Data Availability

The data that support the findings of this study are available from the corresponding author upon reasonable request.

## Abbreviations

The following abbreviations are used in this manuscript:

ACS	Acute Coronary Syndrome
A-IMR	Angio-Based Index of Microcirculatory Resistance
angio-IMR	Angiography-derived hyperemic index of coronary microcirculatory resistance
AS	Aortic Stenosis
AUC	Area Under the Curve
CAD	Coronary Artery Disease
CCS	Chronic Coronary Syndrome
CFD	Computational Fluid Dynamics
CI	Confidence Interval
Cx	Left Circumflex Artery
DM	Diabetes Mellitus
eGFR	Estimated Glomerular Filtration Rate
ESC	European Society of Cardiology
FFR	Fractional Flow Reserve
HT	Hypertension
IMR	Index of Microcirculatory Resistance
IMR_angio_	Angiography-derived index of microcirculatory resistance
IQR	Interquartile Range
LAD	Left Anterior Descending Artery
LVH	Left Ventricular Hypertrophy
MI	Myocardial Infarction
µQFR	Murray law-based Quantitative Flow Ratio
QFR	Quantitative Flow Ratio
PCI	Percutaneous Coronary Intervention
Pd/Pa	Ratio of distal coronary pressure to aortic pressure
RCA	Right Coronary Artery
STEMI	ST-Elevation Myocardial Infarction
